# The transcription factor HBF1 directly activates expression of multiple flowering time repressors to delay rice flowering

**DOI:** 10.1007/s42994-023-00107-7

**Published:** 2023-06-30

**Authors:** Cong Li, Liya Zhang, Xin Wang, Chunsheng Yu, Tao Zhao, Bin Liu, Hongyu Li, Jun Liu, Chunyu Zhang

**Affiliations:** 1 Institute of Crop Science, Chinese Academy of Agricultural Sciences, Beijing, 100081 China; 2 Key Laboratory of South China Agricultural Plant Molecular Analysis and Genetic Improvement and Guangdong Provincial Key Laboratory of Applied Botany, South China Botanical Garden, Chinese Academy of Sciences, Guangzhou, 510650 China; 3 Institute of Nanfan and Seed Industry, Guangdong Academy of Sciences, Guangzhou, 510316 China

**Keywords:** *HBF1*, bZIP transcription factor, *OsWRKY64*, Flowering time, Rice

## Abstract

Flowering time (or heading date) is an important agronomic trait that determines the environmental adaptability and yield of many crops, including rice (*Oryza sativa* L.). Hd3a BINDING REPRESSOR FACTOR 1 (HBF1), a basic leucine zipper transcription factor, delays flowering by decreasing the expression of *Early heading date 1* (*Ehd1*), *Heading date 3a* (*Hd3a*), and *RICE FLOWERING LOCUS T 1* (*RFT1*), but the underlying molecular mechanisms have not been fully elucidated. Here, we employed the hybrid transcriptional factor (HTF) strategy to enhance the transcriptional activity of HBF1 by fusing it to four copies of the activation domain from *Herpes simplex virus* VP16. We discovered that transgenic rice lines overexpressing *HBF1-VP64* (*HBF1V*) show significant delays in time to flower, compared to lines overexpressing *HBF1-MYC* or wild-type plants, via the *Ehd1*–*Hd3a*/*RFT1* pathway, under both long-day and short-day conditions. Transcriptome deep sequencing analysis indicated that 19 *WRKY* family genes are upregulated in the *HBF1V* overexpression line. We demonstrate that the previously unknown gene, *OsWRKY64*, is a direct downstream target of HBF1 and represses flowering in rice, whereas three known flowering repressor genes, *Days to heading 7* (*DTH7*), *CONSTANS 3* (*OsCO3*), and *OsWRKY104*, are also direct target genes of HBF1 in flowering regulation. Taking these results together, we propose detailed molecular mechanisms by which HBF1 regulates the time to flower in rice.

**Supplementary Information:**

The online version contains supplementary material available at 10.1007/s42994-023-00107-7.

## Introduction

The timing of flowering is coordinately controlled by endogenous and environmental factors, such as photoperiod, temperature, nutrient availability, phytohormones, and plant age (Quiroz et al. 2021). As rice is a facultative short-day (SD) plant, photoperiod-mediated flowering (*Oryza sativa* L.) is critical for its regional adaptation and yield. Transcriptional and post-transcriptional components regulating rice photoperiodic flowering have been extensively investigated. Two major pathways with cross-connections have been identified: the evolutionarily conserved *OsGIGANTEA* (*OsGI*)*–Heading date 1* (*Hd1*)*–Heading date 3a* (*Hd3a*)–*RICE FLOWERING LOCUS T 1* (*RFT1*) pathway, analogous to *Arabidopsis* (*Arabidopsis thaliana*) *GI–CONSTANS* (*CO*)–*FLOWERING LOCUS T* (*FT*) pathway; and the rice-specific *Grain number*, *plant height*, *and heading date 7* (*Ghd7*)*–Early heading date 1* (*Ehd1*)*–Hd3a/RFT1* pathway (Zhou et al. 2021). The two pathways are integrated at the level of the florigen genes *Hd3a* and *RFT1* to modulate flowering. Hd3a and RFT1, from the phosphatidylethanolamine-binding protein (PEBP) family, are close homologs of *Arabidopsis* FT (Kojima et al. 2002). Although *Hd3a* and *RFT1* are expressed in leaves, their protein products are delivered to the shoot apical meristem (SAM) through the phloem, where they activate the transcription of downstream floral identity genes and trigger the transition to flowering (Tamaki et al. 2007). They exhibit distinct functions, with Hd3a inducing flowering under SD conditions, while RFT1 promotes flowering under long-day (LD) conditions (Komiya et al. 2009).

Most flowering-related factors act upstream of *Hd3a* or *RFT1*, influencing flowering time by regulating *Hd3a* or *RFT1* expression. *OsGI* encodes an ortholog of *Arabidopsis* GI that activates *Hd1* transcription in the conserved pathway (Huang et al. 2022). The loss of OsGI function results in a late-flowering phenotype under SD conditions, but the effect is weak under LD conditions (Lee et al. 2016). Hd1 exerts a dual function in controlling rice flowering, suppressing flowering under LD conditions but promoting it under SD conditions by regulating *Hd3a* expression (Turck et al. 2008). *Ghd7* and *Ehd1* are specific to monocots and have no clear orthologs in *Arabidopsis* (Zhang et al. 2021). Ehd1 functions as an activator of flowering under both LD and SD conditions and acts upstream of *Hd3a* and *RFT1* independently of Hd1 (Zhou et al. 2021). Ghd7 suppresses *Ehd1* expression, thereby repressing the floral transition (Xue et al. 2008). Furthermore, Ghd7 interacts with Hd1 to form a complex that can repress *Ehd1* transcription through binding to *cis*-regulatory sequences in the *Ehd1* promoter (Zhang et al. 2017b). As these genes are at the core of flowering regulation in rice, their transcript levels offer convenient tools to monitor the state of the flowering pathways in this crop when dissecting its multiple underlying regulatory layers.

In addition to these key flowering genes, multiple flowering regulators have been identified in rice. *Days to heading 7* (*DTH7*, also named *PSEUDO-RESPONSE REGULATOR 37*, *OsPRR37*) encodes a pseudo-response regulator that downregulates *Ehd1* and *Hd3a* expression and results in a late-flowering phenotype under LD conditions. Many European and Asian rice cultivars from higher latitudes harbor nonfunctional *DTH7* alleles and have an early-flowering phenotype (Koo et al. 2013). Independently of the *Ehd1* pathway, another flowering repressor, the CONSTANS-LIKE (COL) protein OsCO3, controls flowering time under SD conditions by negatively regulating the expression of *Hd3a* (Kim et al. 2008). Many transcription factors are also involved in rice flowering regulation. OsWRKY11 (also named semi-dwarf and late flowering 1, Dlf1) acts as a transcriptional activator and regulates flowering by downregulating *Ehd2* expression (Cai et al. 2014). Similarly, OsWRKY104 suppresses *Ehd1* expression and confers a late-flowering phenotype in rice (Zhang et al. 2016).

The basic leucine zipper (bZIP) family of transcription factors (TF) play diverse roles in rice development, including abiotic stress responses, light signal transduction, flower development, pathogen defense, and seed maturation. Little is known, however, about their roles in rice flowering (Zong et al. 2020). OsFD1, the counterpart of *Arabidopsis* FD in rice, forms a florigen activation complex (FAC) with Hd3a and 14-3-3 proteins in the SAM, thus promoting flowering by inducing the expression of downstream MADS-box transcription factor genes (Taoka et al. 2011). Many other bZIP TFs can form alternative FACs by replacing OsFD1 in the complex and regulate flowering. For instance, the bZIP TF Hd3a BINDING REPRESSOR FACTOR 1 (HBF1, also called bZIP42) forms a FAC by substituting for OsFD1, acting as a suppressor of rice flowering (Brambilla et al. 2017). HBF1 physically interacts with Hd3a and *HBF1* overexpression decreased the expression of *Ehd1*, *Hd3a* and *RFT1* in leaves, causing delayed flowering (Brambilla et al. 2017). These studies indicate that Hd3a-mediated transcriptional activation or repression complexes can regulate rice flowering via other bZIP TFs. Our previous study revealed that the bZIP family members ABA RESPONSIVE ELEMENT BINDING FACTOR 1 (OsABF1) and its closest homolog OsbZIP40 suppress the floral transition by activating *OsWRKY104* transcription in a photoperiod-independent manner (Zhang et al. 2016). Recently, bZIP71 was demonstrated to delay flowering by suppressing *Ehd1* expression in rice (Li et al. 2022).

In this study, we explored the molecular mechanisms underlying HBF1-mediated regulation of time to flower in rice. Transcriptome deep sequencing (RNA-seq) analysis showed that many WRKY family genes are upregulated upon overexpression of *HBF1V*, encoding a fusion between HBF1 and four copies of the VP16 activation domain. Among them, we focused here on the flowering repressor *OsWRKY64* as a direct downstream target of HBF1. In addition, we demonstrate that HBF1 can directly activate the transcription of multiple other flowering repressor genes, including *DTH7*, *OsCO3*, and *OsWRKY104*, to inhibit rice flowering through both *Ehd1*-dependent and -independent pathways.

## Results

### Hybrid transcription factor HBF1-VP64 delays flowering time in rice

**Fig. 1:**
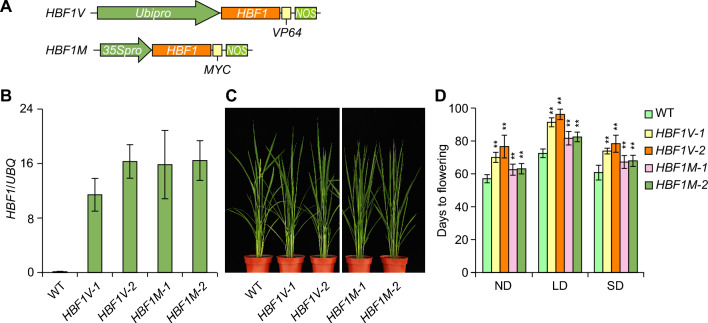
Overexpression of *HBF1V* or *HBF1M* results in the late flowering phenotype. **A** Diagram of the *HBF1V* and *HBF1M* constructs. *HBF1V*, *Ubipro:HBF1-VP64*; *HBF1M*, *35Spro:HBF1-MYC*. **B** Expression of *HBF1* in indicated lines by reverse-transcription quantitative PCR (RT-qPCR). **C** Representative flowering image of indicated genotypes under natural day (ND) conditions in Beijing. **D** Flowering time of each genotype under LD or SD conditions. LD, long-day (14 h light/10 h dark), SD, short day (10 h light/14 h dark). Data were means ± s.d. (standard deviations, *n* = 20). The data of each genotype were compared with wild type (WT, Kita-ake) (Student’s *t* tests, **P < 0.01, *n* = 20)

In the hybrid transcription factor (HTF) system, the sequence encoding VP64 (tetrameric repeats of the VP16 activation domain from *Herpes simplex virus*) is cloned in-frame with the coding sequence of the TF gene of interest. This method has been demonstrated to be effective for the study of TFs in rice and other species (Zhao et al. 2015). In our study, we obtained eight transgenic lines constitutively expressing *HBF1-VP64* in rice under the control of the maize (*Zea mays*) *UBIQUITIN1* promoter (*Ubipro*) through Agrobacterium (*Agrobacterium tumefaciens*)-mediated transformation (Fig. 1A, upper panel); these *Ubipro:HBF1-VP64* lines will be referred to as *HBF1V* thereafter. We established that all positive *HBF1V* lines delay flowering time under natural day (ND, summer in Beijing) conditions, in particular lines *HBF1V-1* and *HBF1V-2*, which we chose for characterization. Reverse-transcription quantitative PCR (RT-qPCR) analysis determined that *HBF1* expression in the *HBF1V-1* and *HBF1V-2* lines is higher than that in wild type (WT, Kita-ake) (Fig. 1B). To precisely assess their flowering time, we grew WT, *HBF1V-1*, and *HBF1V-2* plants under long-day (LD, 14 h light/ 10 h dark), short-day (SD, 10 h light/14 h dark), and ND conditions and counted the number of days from sowing to heading as flowering time. We determined that the *HBF1V-1* and *HBF1V-2* lines both flower significantly later than WT under LD, SD, and ND conditions (Fig. 1C and D).

To elucidate the function of *HBF1* in regulating time to flower, we generated the construct *35Spro:HBF1-MYC* (*HBF1M*) and obtained multiple transgenic lines through Agrobacterium-mediated transformation (Fig. 1A, lower panel). We confirmed the high expression of *HBF1* in these transgenic plants by RT-qPCR; we chose the two independent *HBF1M* overexpression lines *HBF1M-1* and *HBF1M-2*, with similar *HBF1* expression levels to *HBF1V* lines (Fig. 1B). Although the overexpression of *HBF1M* significantly delayed flowering time, this effect was less pronounced than that seen for *HBF1V* lines under LD, SD, and ND conditions, indicating that *HBF1V* exerts a stronger flowering repressor activity than *HBF1M* in these transgenic plants (Fig. 1C and D). We conclude that HBF1 is a negative regulator of rice flowering time, with HBF1V exerting a stronger delaying effect on flowering than HBF1M.

**Fig. 2:**
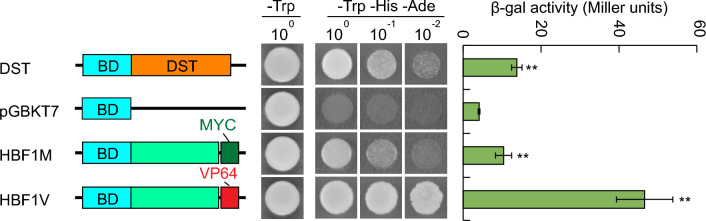
Yeast transcriptional activation activity assay of HBF1 protein. (Left panel) Diagrams of yeast bait constructs containing HBF1-MYC (HBF1M) and HBF1-VP64 (HBF1V). DNA-binding domain of yeast GAL4 (BD) and BD fused with DST were used as negative and positive controls respectively. (Middle panel) Plate auxotroph assays showed transcriptional activation activity of each protein. (Right panel) Quantitative the transcriptional activation activity of each protein. β-galactosidase activity data were means of ± s.d. (Student’s *t* tests, **P < 0.01, *n* = 3)

To explore the quantitative difference in flowering time shown by the *HBF1V* and *HBF1M* lines, we examined the transcriptional activity of HBF1M and HBF1V proteins in a yeast (*Saccharomyces cerevisiae*) transcriptional activation assay. To this end, we fused HBF1V or HBF1M to the DNA-binding domain of yeast GAL4 (BD) and transformed the encoding constructs individually into a yeast strain. We observed that HBF1 exhibits transcriptional activation activity, whereas the negative control (the GAL4 BD alone) did not. Moreover, HBF1V had stronger transcriptional activation activity than HBF1M (Fig. 2), indicating that the addition of the VP64 domain markedly increased the transcriptional activation activity of HBF1. Combined with the flowering phenotype reporter above, these results indicate that the stronger transcriptional activation activity of HBF1V is likely responsible for the later-flowering phenotype of *HBF1V* transgenic lines relative to *HBF1M* lines.

### HBF1 delays flowering partially through the *Ehd1* pathway

**Fig. 3:**
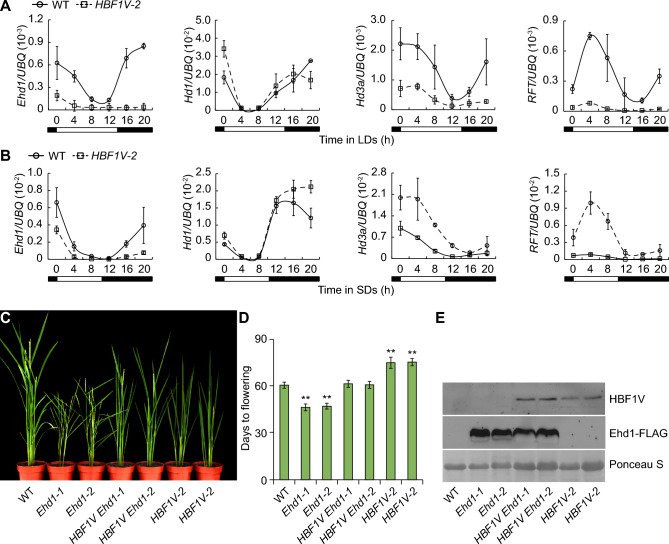
HBF1 delays floral initiation incompletely dependent on *Ehd1* pathway. **A**, **B** Expression analysis of *Ehd1*, *Hd3a*, *RFT1* and *Hd1* in WT and *HBF1V-2* plants. Plants were grown under LD (**A**) or SD (**B**) conditions for 4 weeks, newly expended leaves were collected every 4 h from the beginning of the light period for RNA extraction. Three biological replicates were performed, and *Ubiquitin* (*UBQ)* was used as internal control. Data were means ± s.d. (*n* = 3). **C** Phenotypes of indicated genotypes at heading stage grown under ND conditions. *Ehd1-1* and *Ehd1-2* were generated by transformation of the *Ubipro:Ehd1-FLAG* plasmids into wild-type. *HBF1V Ehd1-1* and *HBF1V Ehd1-2* were two independent T1 lines generated by transformation of *Ubipro:Ehd1-FLAG* construct into the *HBF1V-2* homozygotes line. **D** Statistical analysis of flowering time in indicated genotypes. Data of each genotype were compared with WT (Student’s *t* tests, **P < 0.01, *n* = 20). **E** Western blot detects HBF1V or Ehd1-FLAG in each genotype by using anti-VP16 or anti-FLAG antibody respectively. The ponceau S staining band of Rubisco large subunit was used as loading control

A previous study showed that *HBF1* transcripts are highly abundant in the SAM and leaves (Brambilla et al. 2017). We monitored *HBF1* expression in various tissues of Kita-ake using RT-qPCR, which revealed that *HBF1* is expressed in all tissues, with high expression in leaves and sheaths (Fig. S1A). Consistent with these results, we detected strong β-glucuronidase (GUS) staining in the leaves, sheaths, stems, roots and panicles of transgenic plants harboring the *HBF1pro:GUS* reporter construct (Fig. S1B). As the diurnal expression pattern of flowering genes is critical to their role, we measured the expression of four key flowering genes (*Ehd1*, *RFT1*, *Hd3a*, and *Hd1*) in leaves over one diurnal cycle by collecting samples every 4 h. We determined that the expression of *Ehd1*, *RFT1*, and *Hd3a* is significantly downregulated in the *HBF1V*-*2* line compared to WT under LD and SD conditions, while *Hd1* expression showed little difference from WT (Fig. 3A and B). We examined the expression of another 12 flowering-time-related genes in rice, but detected no significant differences between WT and *HBF1V-2* under either LD or SD conditions (Fig. S2 and S3). These results demonstrate that HBF1V delays flowering time through lowering the expression of *Ehd1*, *Hd3a*, and *RFT1* in leaves.

To analyze the genetic relationship between *HBF1* and *Ehd1*, we generated the *Ubipro*:*Ehd1-FLAG* construct and transformed it into WT and *HBF1V-2*. We obtained several transgenic lines, from which we chose four overexpression lines, named *Ehd1*-*1*, *Ehd1-2*, *HBF1V Ehd1-1*, and *HBF1V Ehd1-2*, for characterization. We observed a late-flowering phenotype for *HBF1V Ehd1* overexpression lines and WT compared to *Ehd1* overexpression lines under LD conditions (Fig. 3C and D). We confirmed the accumulation of the respective tagged proteins in each transgenic line by immunoblotting with anti-FLAG and anti-VP64 antibodies (Fig. 3E). Thus, molecular and genetic lines of evidence indicate that HBF1 delays flowering in a manner partially dependent on the *Ehd1* pathway.

### HBF1 inhibits rice flowering by directly activating *OsWRKY64* transcription

**Fig. 4:**
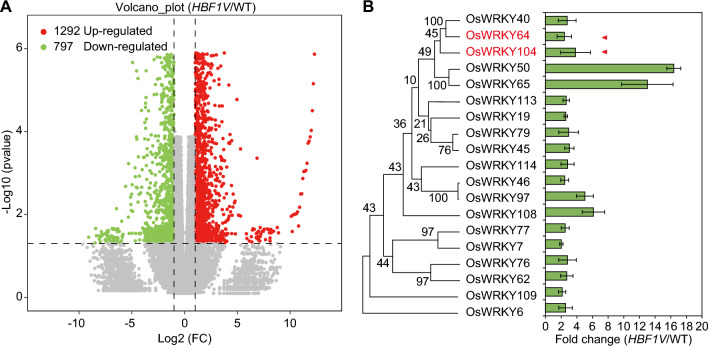
The 19 *WRKY* genes are up-regulated significantly in RNA-seq data. **A** Volcano plot displays differentially expressed genes between WT and *HBF1V-2* (Fold Change (FC) > 2, *P* value < 0.01, and False Discovery Rate (FDR) < 0.01). WT and *HBF1V-2* transgene lines were grown under continuous light in plant growth chamber at 28 °C for 4 weeks. Ten newly expended leaves were collected to extract total RNA and three biological replicates were performed to verify the RNA-sequence results. **B** Phylogenetic tree of the 19 WRKY genes that up-regulated in *HBF1V-2* (Left panel). Protein sequences were downloaded from the MSU Rice Genome Annotation Project (http://rice.plantbiology.msu.edu/analyses_search_locus.shtml) databases and then used for Neighbor-joining phylogenetic analysis (MEGA5.2). Fold change analysis of 19 WRKY genes in HBF1V compared with those in WT (Right panel)

Although HBF1 was previously shown to bind to the *Ehd1* promoter (Brambilla et al. 2017), *Ehd1* was expressed at lower levels in the *HBF1* overexpression lines relative to WT (Fig. 3A). Since HBF1 is proposed to work as a transcriptional activator, we suspected that other downstream genes of HBF1 may function upstream of *Ehd1*. To uncover the downstream targets of HBF1, we performed RNA-seq using leaves of 4-week-old WT and *HBF1V-2* seedlings. We identified differentially expressed genes (DEGs) in *HBF1V* seedlings relative to WT with cutoffs of fold-change ± 2, *P*-value < 0.01, and false discovery rate (FDR) < 0.01. A volcano plot of these DEGs indicated that 1292 genes are upregulated and 797 genes are downregulated in *HBF1V* seedlings compared to WT (Fig. 4A and Table S2). We randomly selected 20 upregulated DEGs for validation by RT-qPCR (Fig. S4). A careful inspection of the RNA-seq data revealed that 19 out of 129 WRKY family genes in *japonica* rice are upregulated in *HBF1V-2* seedlings (Table S3). A phylogenetic analysis of the proteins encoded by all upregulated *WRKY* genes in *HBF1V-2* using MEGA5.2 showed that OsWRKY64 is the closest homolog to OsWRKY104 (Fig. 4B). Although *OsWRKY104* has been identified as a direct target of OsABF1 (Zhang et al. 2016), the function of *e* genes in flowering regulation remains elusive due to the lack of the loss-of-function mutants of these genes, which prompted us to investigate the role of *OsWRKY64* in the regulation of flowering.

**Fig. 5:**
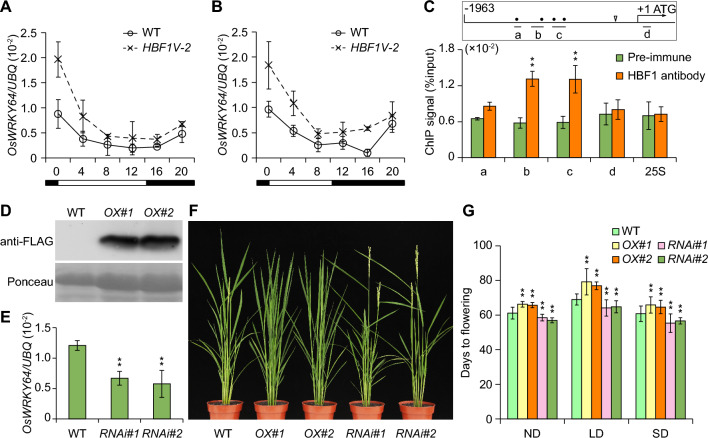
*OsWRKY64* is direct target of HBF1. **A**, **B** Expression analysis of *OsWRKY64* in each genotype under LD (A) or SD (B) conditions using RT-qPCR. Three biological replicates were performed and the *UBQ* were used as internal control. Data were means ± s.d. (Student’s *t* tests, **P < 0.01, *n* = 3). **C** Verification of the binding sites of HBF1 in *OsWRKY64* promoter by ChIP-qPCR. ChIP samples were collected from Kita-ake plants and precipitated with anti-HBF1 antibody. qPCR data were normalized to the input signal. The binding of HBF1 to 25S rDNA was used as negative control. Data were means ± s.d. (*n* = 3, Student’s *t* tests, **P < 0.01). The promoter diagram of *OsWRKY64* is shown in the boxes. The short lines above a, b or c represent the distribution of PCR fragments on promoter region. The dots indicate the position of ACGT core sequence, triangle indicates the position of translation start site (TSS), + 1 represents the position of start codon ATG. **D** Western blot analysis of OsWRKY64-FLAG in indicated genotypes by using anti-FLAG antibody. The ponceau S staining bands of Rubisco large subunit was used as loading control. **E** RT-qPCR analysis of *OsWRKY64* expression in indicated lines. Data were means ± s.d. (Student’s *t* tests, **P < 0.01, *n* = 3). **F** Flowering image of each genotype under ND conditions in Beijing. *OX*, *Ubipro:OsWRKY64*-*FLAG*; *RNAi*, *OsWRKY64 RNAi*. **G** Statistical analysis of flowering time in each genotype under LD, SD, and ND conditions, data were means ± s.d. (Student’s *t* tests, **P < 0.01,* n* ≥ 12)

RT-qPCR analysis verified that *OsWRKY64* transcript abundance is higher in *HBF1V-2* seedlings than in WT under both LD and SD conditions (Fig. 5A and B). Consistent with this finding, we identified three ABRE (ABA-response element, ACGT-containing sequences) *cis*-elements, known binding elements for bZIP TFs, within the *OsWRKY64* promoter sequence. We therefore investigated whether HBF1 might bind to the *OsWRKY64* promoter by performing an in vivo chromatin immunoprecipitation followed by quantitative PCR (ChIP-qPCR) experiment using WT Kita-ake seedlings and an anti-HBF1 antibody (Fig. 5C). Indeed, we observed robust binding of HBF1 to the b and c sites in the *OsWRKY64* promoter (Fig. 5C). Transient expression assays in *Nicotiana benthamiana* leaves revealed that HBF1 can increase firefly luciferase (LUC) activity derived from a *OsWRKY64pro:LUC* reporter construct (Fig. S5), suggesting that HBF1 positively regulates *OsWRKY64* transcription. To explore the function of OsWRKY64 in rice, we generated *OsWRKY64* overexpression and RNA interference (RNAi) lines. We chose the two independent transgenic *Ubipro:OsWRKY64*-*FLAG-*OX (*OX#1* and *OX#2*) lines with increased accumulation of OsWKRY64-FLAG protein, as well as two *OsWRKY64-*RNAi lines with lower *OsWRKY64* transcript levels, for further analysis (Fig. 5D and E). The two *OsWRKY64* overexpression lines flowered late, while the two RNAi lines flowered early, compared to WT when grown under LD, SD, or ND conditions (Fig. 5F and G). These results suggest that *OsWRKY64* is a direct target gene of HBF1 and inhibits flowering in rice.

### HBF1 is a transcriptional activator of *DTH7*, *OsCO3*, and *OsWRKY104*

**Fig. 6:**
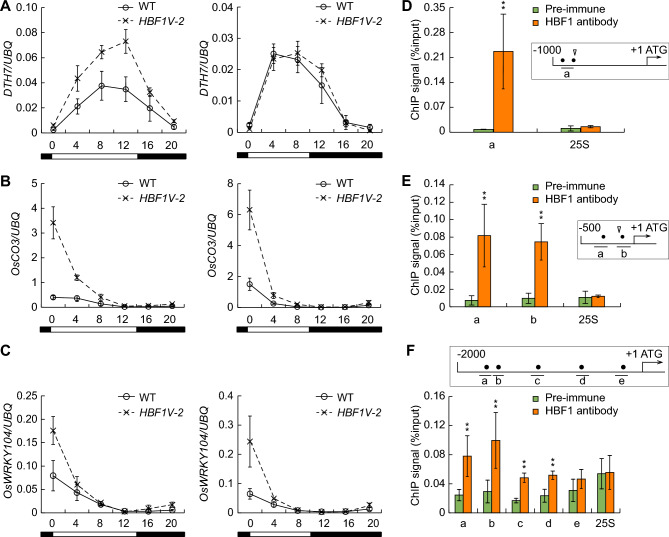
HBF1 is transcriptional activator of *DTH7*, *OsCO3*, and *OsWRKY104*. **A**–**C,** RT-qPCR analysis of the dynamic expression of *DTH7* (**A**), *OsCO3* (**B**), and *OsWRKY104* (**C**) in each genotype under LD or SD conditions. Three biological replicates were performed and *UBQ* was used as internal control. **D**–**F** Verification of HBF1 binding sites in *DTH7* (**D**), *OsCO3* (**E**), and *OsWRKY104* (**F**) promoters by ChIP-qPCR. ChIP samples were collected from Kita-ake plants and precipitated with anti-HBF1 antibody. qPCR data were normalized to the input signal. The binding of HBF1 to 25S rDNA was used as negative control. Data were means ± s.d. (*n* = 3, Student’s *t* tests, **P < 0.01). The promoter diagrams of *DTH7, OsCO3*, and *OsWRKY104* were shown in the boxes. The short lines under or above a, b or c represent the distribution of PCR fragments, and the ACGT core sequence were indicated by black dots. Triangle indicates the position of TSS, + 1 represents the first nucleotide of start codon ATG

From the RNA-seq dataset, we noticed that three flowering repressors, *DTH7*, *OsCO3*, and *OsWRKY104*, were upregulated in the *HBF1V-2* line (Table S2). To explore this observation, we conducted an RT-qPCR analysis over a diurnal time course under LD and SD conditions. The expression of *OsCO3* and *OsWRKY104* was upregulated in *HBF1V-2* compared to WT during the daytime under both LD and SD conditions, whereas *DTH7* was upregulated in LD only but not in SD, suggesting that HBF1 might act as a direct activator of *DTH7*, *OsCO3*, and *OsWRKY104* to regulate flowering time in rice (Fig. 6A–C). To test this hypothesis, we inspected the promoter sequences of *DTH7*, *OsCO3*, and *OsWRKY104*, which revealed ABRE (ACGT-containing sequences) *cis*-elements (Fig. 6D–F, insets). We performed ChIP-qPCR to test the direct interactions between HBF1 and these three promoters. We established that HBF1 can specifically bind to all three promoters, but not to the *25S* rDNA locus, which was used as negative control (Fig. 6D–F). Transient expression assays in *N. benthamiana* leaves revealed that the presence of HBF1 results in higher LUC activity from the *DTH7pro:LUC*, *OsCO3pro:LUC*, and *OsWRKY104pro:LUC* reporter constructs (Fig. S5), suggesting that HBF1 can positively regulate their transcription. These results support the notion that HBF1 directly activates the transcription of *DTH7*, *OsCO3*, and *OsWRKY104* in the context of flowering regulation.

## Discussion

### HBF1V confers an enhanced function to HBF1 in rice flowering

Although bZIP TFs are important in various biological programs, especially in abiotic stress tolerance, in rice, little is known about their involvement in flowering. HBF1/OsbZIP42 was reported to form transcriptional repressive complexes with Hd3a to regulate the expression of *Ehd1*, *Hd3a*, and *RFT1* in leaves (Brambilla et al. 2017). However, few direct target genes of HBF1 have been identified in rice beside *Ehd1*. In this study, we demonstrated using the HTF strategy that HBF1 represses flowering in rice (Zhao et al. 2015). The VP64 fragment fused to HBF1 enhanced its transcriptional activity; *HBF1V* lines exhibited a greater delay in their flowering compared to *HBF1M* and WT, indicating that the HTF strategy was effective in enhancing the function of HBF1 in flowering regulation of rice. Our study also confirmed that the HTF strategy is a very effective method to study TFs (Zhang et al. 2016).

### HBF1 regulates flowering through *Ehd1*-dependent and -independent pathways

**Fig. 7:**
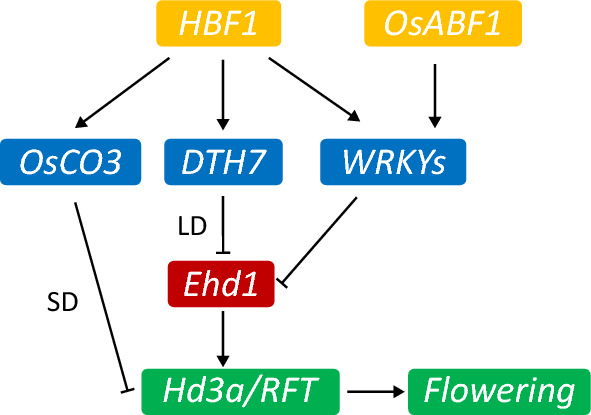
A working model for *HBF1* in rice flowering. HBF1 directly activates three flowering repressors to promote rice flowering time through *Ehd1*-dependent and *Ehd1*-indenpent pathway under LD and SD, respectively. In addition, HBF1 and OsABF1 co-target WRKYs to regulate flowering time in rice

*Ehd1* encodes a B-type response regulator that promotes flowering by inducing the expression of *Hd3a* and *RFT1*. In this study, molecular and genetic evidence support the idea that HBF1 partially depends on the *Ehd1* pathway to repress flowering time, which is consistent with a previous study (Brambilla et al. 2017). Indeed, we showed two flowering repressor genes, *DTH7* and *OsCO3*, to be direct targets of HBF1 (Fig. 7). DTH7 mainly acts as a suppressor of *Ehd1* in an LD-dependent manner (Gao et al. 2014). The wild-type background used in our study, Kita-ake, is thought to harbor nonfunctional alleles of several flowering genes, including *DTH7*, with the polymorphisms in *DHT7* causing changes in conserved amino acids that may affect DTH7 function. Notably, *DTH7* transcript levels in Kita-ake were not lower than those of cultivars carrying a fully functional *DTH7* allele (Gao et al. 2014), indicating that Kita-ake may carry a weak *DTH7* allele rather than a loss-of-function allele. Therefore, our study raises the possibility that *DTH7* is a direct target of HBF1 in rice to regulate flowering. OsCO3 regulates the expression of *Hd3a* but not *Ehd1*, to delay flowering under SD conditions (Kim et al. 2008), offering evidence that HBF1 functions in rice flowering independently of the *Ehd1* pathway. Additionally, our study suggests that HBF1 regulates rice flowering through distinct pathways under LD and SD conditions.

### *WRKY* genes mediate the regulation of flowering time in rice

In rice, few WRKY TFs have been reported to participate in flowering time, with the exceptions of *OsWRKY11* and *OsWRKY104*. However, the function of *WRKY* genes in flowering remains elusive due to the lack of the loss-of-function mutants. In our study, RNA-seq analysis indicated that 19 *WRKY* family genes were upregulated in *HBF1V-2* plants. Among these genes, we established that *OsWRKY64* transcription is directly activated by HBF1 and represses rice flowering. Knockdown of *OsWRKY64* by RNA interference significantly promoted flowering time in rice. Therefore, our results provide evidence in support for the important roles of *WRKY* members in rice flowering.

### The relationship between HBF1 and OsABF1 in rice flowering

bZIP TFs can form homo- or heterodimers to bind to their cognate DNA sequence. In our previous work, we showed that HBF1 interacted with OsABF1 in Arabidopsis protoplasts (Fig. S6A), indicating that HBF1 and OsABF1 may form a heterodimer to target their candidate genes. In the RNA-seq dataset generated here, 45% of all genes upregulated in *HBF1V* were also more highly expressed in *OsABF1V* than in WT (Fig. S6B, Table S4), suggesting that HBF1 and OsABF1 might regulate the expression of the same genes during plant development. During rice flowering, both HBF1 and OsABF1 bound to the promoters of *OsCO3*, *DTH7*, *OsWRKY64*, and *OsWRKY104* (Fig. S6C). The transcript level of *OsWRKY64* was upregulated in *OsABF1V* under SD but not LD conditions (Fig. S6D). However, the expression of *DTH7* and *OsCO3* was unchanged in *OsABF1V* transgenic lines (Fig. S6E and F), suggesting that HBF1 may target specific flowering-related genes, or share the same set of target genes as OsABF1 to regulate flowering time in rice.

## Materials and methods

### Generation of transgenic rice

To generate *HBF1V* overexpression lines, *HBF1* cDNA was inserted into pBCV (constructed by our lab) expression vector using the Gateway cloning system (Zhao et al. 2015). For *HBF1M* overexpression lines, the *HBF1* cDNA driven by 35S promoter was cloned into binary vector pCAMBIA1390 (reconstructed by our lab) using the Gateway cloning system. To generate *Ehd1* and *OsWRKY64* overexpression lines, their cDNA was inserted into the pHCF (constructed by our lab) vector at *Pst*I site using the Infusion system (Clontech) (Zhang et al. 2016). To generate *OsWRKY64*-*RNAi* plants, a 266-bp fragment of the *OsWRKY64* gene was inserted into the pANDA vector using the Gateway cloning system. The constructs were introduced into Kita-ake rice (*Oryza sativa japonica*) or indicated background by *Agrobacterium tumefaciens-*mediated transformation (Hiei et al. 1994).

### Growth conditions

For RT-qPCR, RNA-seq, and ChIP assays, seeds of wild-type rice and transgenic lines were germinated for 2 d on wet filter paper in petri dishes at 37 °C. The uniformly germinated seeds were picked up and sown in bottomless 96-well plates and hydroponically grown (distiller water with 1/10 Murashige and Skoog). To investigate the flowering phenotypes, all plants were grown under natural day (ND) conditions in Beijing (39°54′N, 116°23′E), China, or under long-day (LD) (14 h light, 28 °C; 10 h dark, 24 °C) conditions or short-day (SD) (10 h light, 28 °C; 14 h dark, 24 °C) conditions in plant growth chambers.

### Yeast transcriptional activation activity assay

To test the transcriptional activation activity, the indicated CDS was fused with GAL4 DNA-binding domain in the pGBKT7 vectors using Infusion system (Clotech) and transformed into the yeast strain *AH109*. The empty vector (BD) and BD-DST vector were used as negative and positive controls, respectively. Measurement of the *β*-galactosidase activity and the colony-lift filter assay were performed according to the Yeast Protocols Handbook (Clontech) using chlorophenol red-*β*-d-galactopyranoside (CPRG, Roche Biochemical) or X-gal (Inalco, Cat.# 1758-0300) as the substrate.

### Chromatin immunoprecipitation (ChIP) assay

The *HBF1V-2* or WT plants under continuous light (CL) were used for ChIP assays. ChIP was performed as described previously (Zhang et al. 2016). Briefly, 3 g of leaves from 4-week-old seedlings were cross-linked by 1% formaldehyde under vacuum for 15 min twice. Then the samples were ground to powder in liquid nitrogen prior to isolating chromatin. After sonicated, the chromatin complexes were incubated with anti-VP16 or anti-HBF1 antibody as described. The precipitated DNA was recovered in water for quantitative real-time PCR. The enrichment value was normalized to that of input DNA (% of input).

### RNA-seq and data analysis

WT and *HBF1V-2* lines were cultivated under continuous light at 28 °C for 4 weeks in plant growth chamber. Ten latest fully expended leaves of each genotype were collected for total RNA extraction and three biological replicates were performed. The sequencing library was constructed following the manufacturer`s instructions, and then sequenced with Illumina HiSeq 2000 at ANOROAD company. Clean reads were mapped to the *O.* ssp. *japonica* genome reference by TopHat. The differentially expressed genes were analyzed by Cuffdiff (q < 0.05) based on FPKM (fragments per kilobase of exon model per million mapped fragments). Differentially expressed genes were defined as those with fold changes ≥ 2 or ≤ 0.5. The volcano plot and venn diagram were analyzed through Omicshare platflom from Gene Denovo Biotechnology Co. (https://www.omicshare.com/).

### Gene expression analyses

To test the mRNA expression of flowering-associated genes in a time course manner under LDs or SDs, plants were grown for 4 weeks and samples were collected every 4 h from the beginning of the light period. RNA was isolated using TRIZOL (Invitrogen) and treated with DNase I (Invitrogen). The cDNA was synthesized from 3.0 μg total RNA using TransScript^®^ II One-Step gDNA Removal and cDNA Synthesis SuperMix (TransGen Biotech). LightCycler 480 SYBR Green I Master (Roche) was used for the quantitative PCR reaction.

### Immunoblot analyses

The anti-HBF1 polyclonal antibody was generated by inoculating rabbits with TF-His- HBF1 recombination protein (Bio-med). The anti-VP16 and anti-OsABF1 polyclonal antibodies were generated in previous study (Zhang et al. 2016). To extract the total protein for immunoblot, the young leaves were ground in liquid nitrogen and mixed with 5** × **SDS-PAGE loading buffer [250 mM Tris (pH 6.8), 10% (w/v) SDS, 0.5% (w/v) bromphenol blue, 50% (v/v) glycerol, 5% (v/v) 2-mercaptoethanol], boiled for 5 min, and spun at 12,000 rpm for 5 min at room temperature. The supernatants were fractioned by 10% SDS-PAGE, and the membrane was probed with the indicated antibody.

### GUS histochemical staining

To obtain *HBF1pro*:*GUS* transgenic plants, a 2221 bp promoter region of *HBF1* was amplified from the genome of Nipponbare, and inserted into the *Hind*III and *BamH*I sites of the pCAMBIA3301-GUS vector. GUS histochemical staining assays were performed according to the previous method (Zou et al. 2015).

### BiFC assays

The *HBF1* or *OsABF1* coding sequences were cloned into the pSPYNE (R) or the pSPYCE (MR) vector (Zou et al. 2015). The vectors were co-transformed into *Arabidopsis* mesophyll protoplasts and incubated overnight before observation. Fluorescence signals were visualized using a Leica TCS-SP4 confocal microscope.

### Transient expression assay

To generate the *OsWRKY64pro:LUC*, *DTH7pro:LUC*, *OsCO3pro:LUC*, and *OsWRKY64pro:LUC* reporter constructs, ~ 2 kb promoters of these genes were cloned into the pGreenII 0800-LUC vector. The *Renilla Luciferase* (*REN*) gene under the control of 35S promoter in the pGreenII 0800-LUC vector was used as the internal control. The coding region of HBF1 was cloned into pGreen-35S:GFP vector to produce *35Spro: HBF1-GFP* construct and used as an effector. These effector and reporter or the control was transformed individually into *Agrobacterium tumefaciens* strain GV3101. GV3101 cells harboring the indicated constructs were mixed at a ratio of 1:1 and introduced into *N. benthamiana* leaves. The LUC and REN activities were measured using the Dual-Luciferase Reporter Assay System under the manufacturers’ instructions. The LUC/REN ratio was presented with three biological replicates.

### Primers and accession numbers

All the primers in this study were listed in Table S1. Sequence data from this article can be found in the MSU Rice Genome Annotation Project (http://rice.plantbiology.msu.edu/analyses_search_locus.shtml) databases (Kawahara et al. 2013) under the following accession numbers: *HBF1* (*LOC_Os05g41070*), *OsABF1* (*LOC_Os01g64730*), *Ehd1* (*LOC_Os10g32600*), *Hd1* (*LOC_Os06g16370*), *Hd3a* (*LOC_Os06g06320*), *RFT1* (*LOC_Os06g06300*), *DTH7* (*LOC_Os07g49460*), *OsCO3* (*LOC_Os09g06464*), *OsWRKY104* (*LOC_Os11g02520*), and *OsWRKY64* (*LOC_Os12g02450*).

### Supplementary Information

Below is the link to the electronic supplementary material.

Supplementary file1 (XLSX 22 KB)

Supplementary file2 (XLSX 285 KB)

Supplementary file3 (XLSX 12 KB)

Supplementary file4 (XLSX 122 KB)

Supplementary file5 (DOCX 1177 KB)

## Data Availability

All relevant data are within the manuscript and its Supplementary files.
