# Supplementary material for: The transcription factor HBF1 directly activates expression of multiple flowering time repressors to delay rice flowering
Source: aBIOTECH. 2023 Jun 30;4(3):213–23. doi: 10.1007/s42994-023-00107-7 (PMC10638126; doi:10.1007/s42994-023-00107-7)
Supplement: Supplementary file 5 — Supplementary file5 (DOCX 1177 KB) [file 42994_2023_107_MOESM5_ESM.docx]

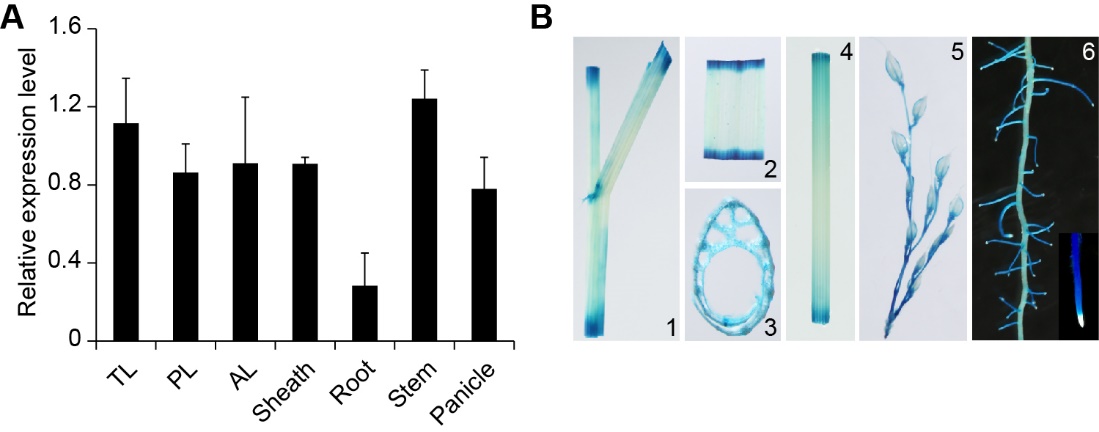


**Fig. S1** Expression pattern of *HBF1*. **A,** Transcript level analysis of *HBF1* in various organs by RT-qPCR (means ± s.d., n = 3). TL, tailender leaf at 4-week stage; PL, penultimate leaf at 4-week stage; AL, antepenult leaf at 4-week stage; Sheath, sheath at 4-week stage; Root, root at 4-week stage. **B,** Histochemical staining of *HBF1pro:GUS* plants in different tissues including shoot (1), leaf (2), sheath (3), stem (4), panicle (5), root (6).


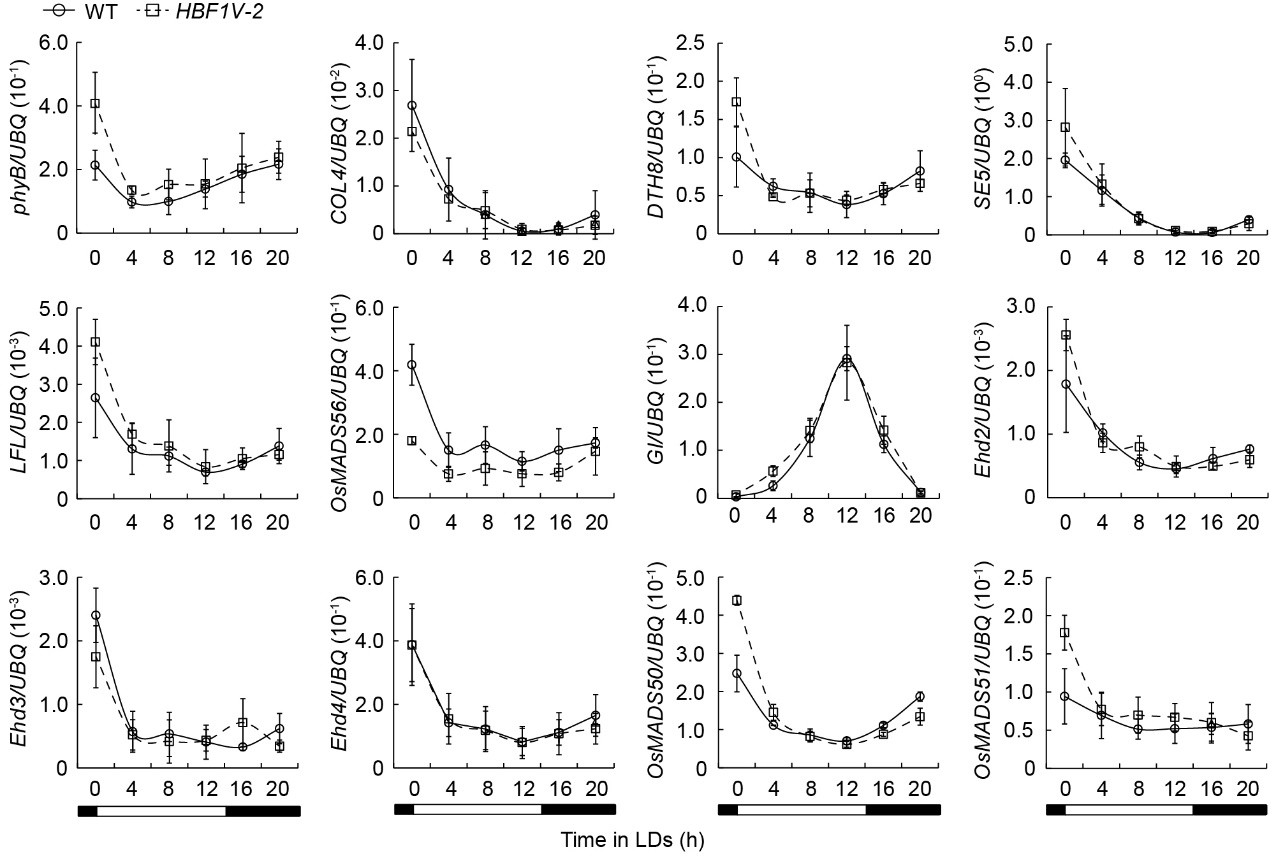


**Fig. S2** RT-qPCR analysis of flowering-associated genes in each genotype under LD conditions. Plants were grown in LD conditions for 4 weeks. Samples were collected every 4 hours from the beginning of light period. Three biological replicates were performed and *UBQ* was used as internal control. Data were means ± s.d. (*n* = 3).


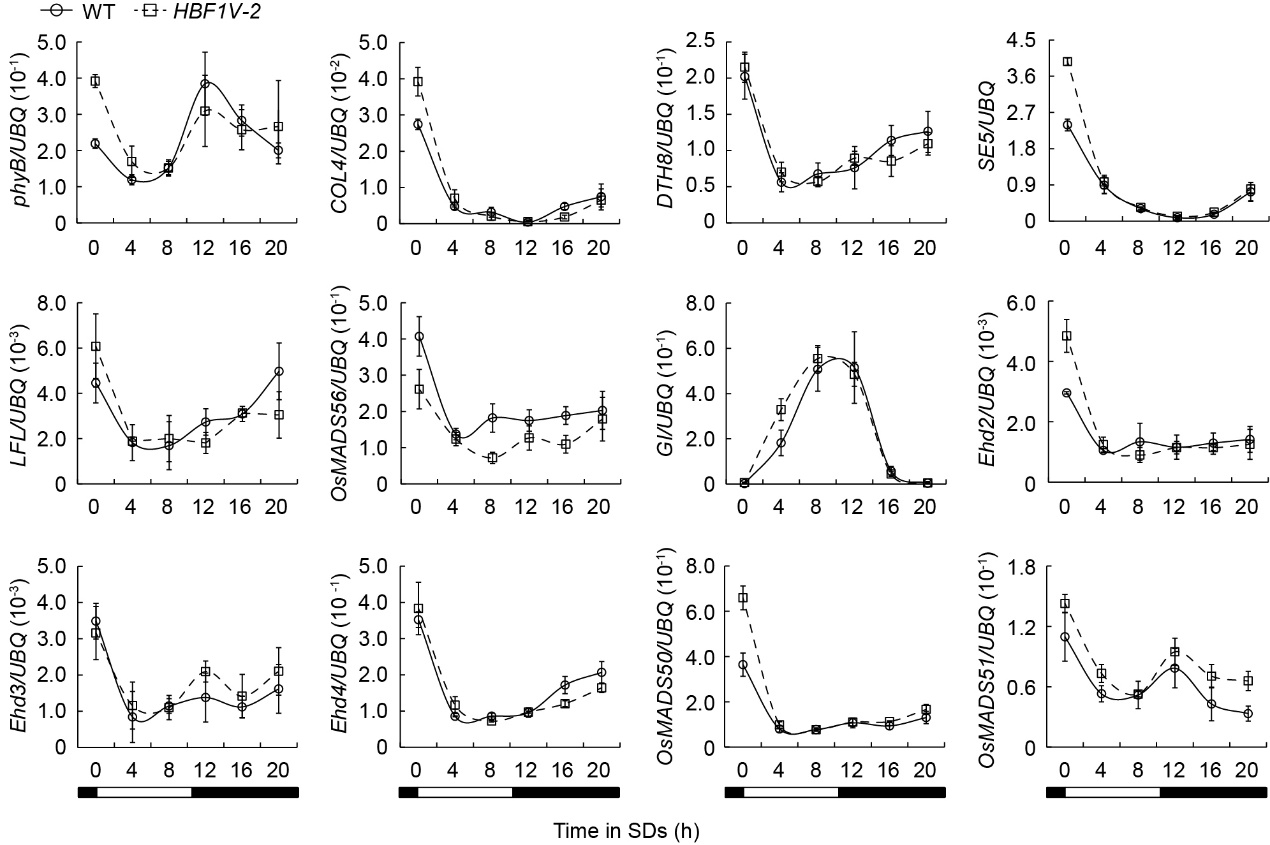


**Fig. S3** RT-qPCR analysis of flowering-associated genes in each genotype under SD conditions. Plants were grown in SD conditions for 4 weeks. Samples were collected every 4 hours from the beginning of the light period. Three biological replicates were performed and *UBQ* was used as internal control. Data were mean ± s.d. (*n* = 3).


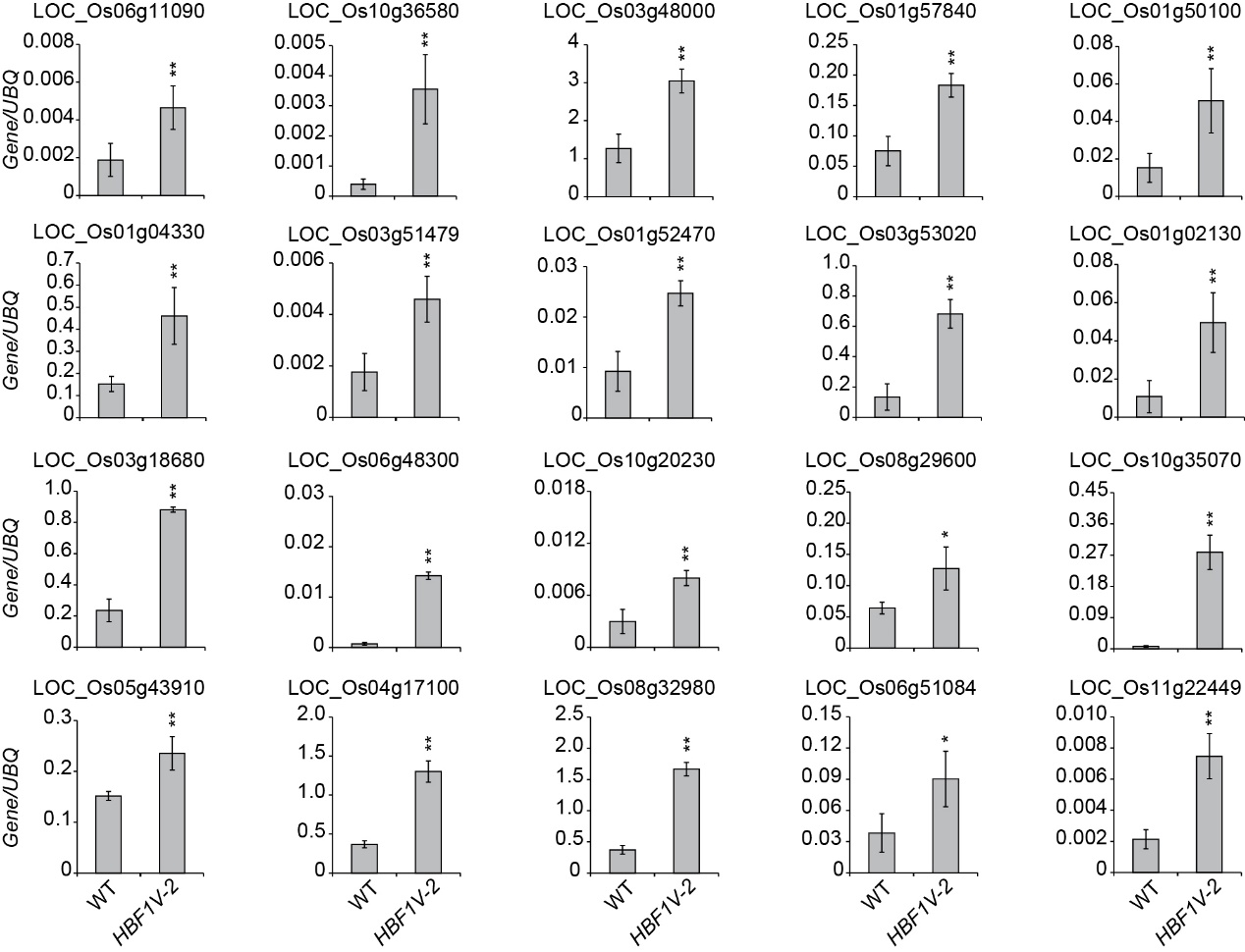


**Fig. S4** Verification of RNA-seq results by RT-qPCR. Twenty genes with increased expression in *HBF1V-2* were selected for RT-qPCR analysis. WT and *HBF1V-2* lines were cultivated under continuous light at 28℃ for 4 weeks in plant growth chamber. Data were means ± s.d. (Student’s *t* tests, **P* < 0.05, ***P* < 0.01, *n* = 3).


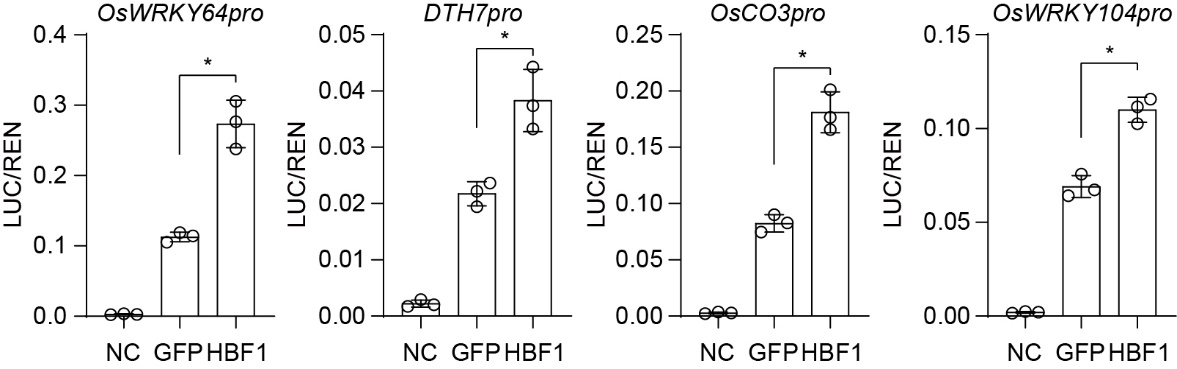


**Fig. S5** Transient expression assay indicating that the expression of *OsWRKY64*, *DTH7*, *OsCO3*, and *OsWRKY104* is regulated by HBF1. Either the reporter or the relevant empty vector (negative control, NC) was co-transformed with the effector or the relevant empty vector (GFP) into tobacco leaves. The activity was calculated by the ratio of LUC to REN. Data were means ± s.d. (Student’s *t* tests, **P* < 0.01, *n* = 3).


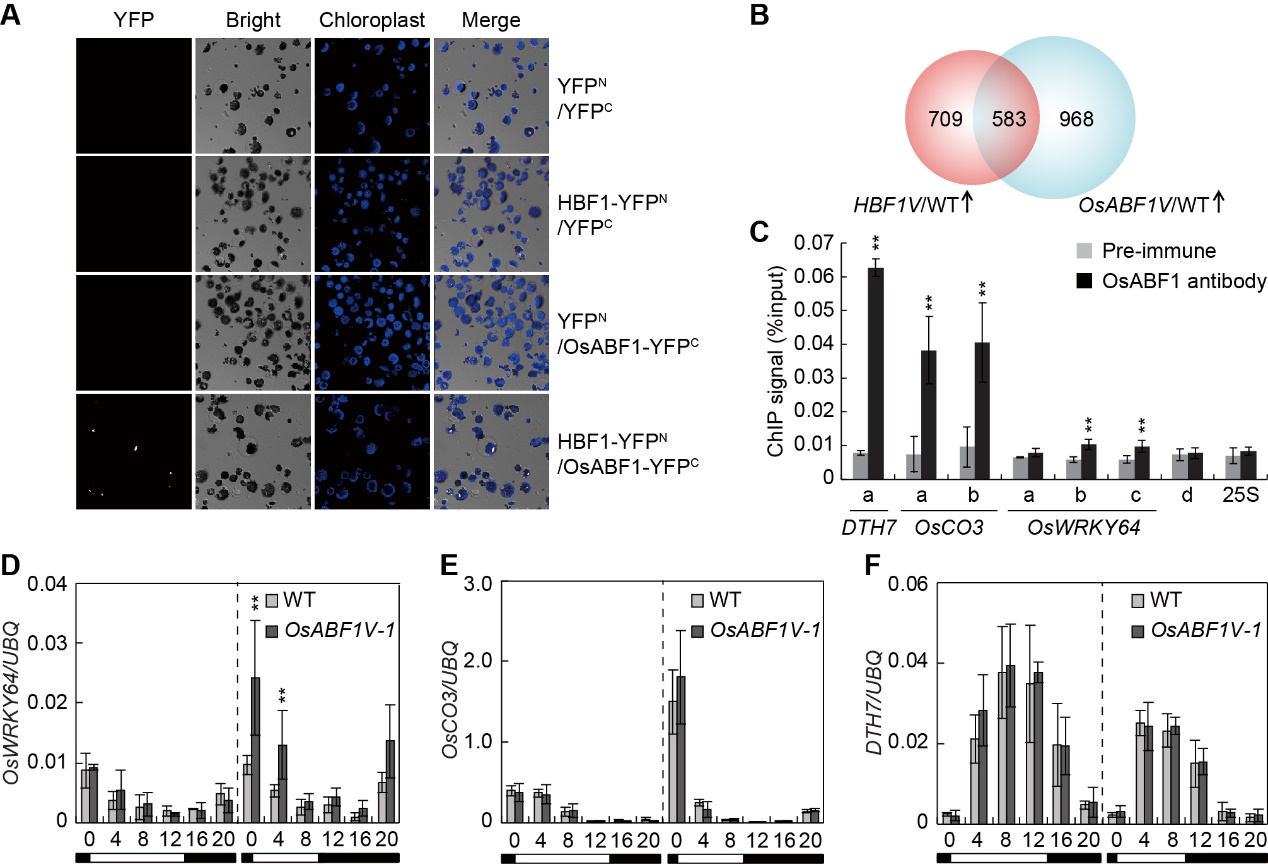


**Fig. S6** The relationship between HBF1 and OsABF1. **A,** HBF1 interacts with OsABF1 in Arabidopsis protoplast. **B,** Venn diagram showing the number of up-regulated genes in *HBF1V* and/or *OsABF1V* transgenic lines. Arrow means up-regulated. **C,** Verification of the direct binding of OsABF1 to the promoters of *DTH7*, *OsCO3*, and *OsWRKY64* by ChIP-qPCR analysis. Data were means ± s.d. (*n* = 3, Student’s *t* tests, ***P* < 0.01). **D-F,** RT-qPCR analysis of the dynamic expression pattern of *DTH7* (D), *OsCO3* (E), and *OsWRKY64* (F) in WT, *OsABF1V-1* transgenic lines under LDs (Right) or SDs (Left). The means ± s.d. (*n* = 3) were shown.
